# A multinational investigation of healthcare needs, preferences, and expectations in supportive cancer care: co-creating the LifeChamps digital platform

**DOI:** 10.1007/s11764-022-01289-7

**Published:** 2022-11-11

**Authors:** Rebecca Marshall-McKenna, Grigorios Kotronoulas, Emmanouil Kokoroskos, Andrea Gil Granados, Panagiotis Papachristou, Nikolaos Papachristou, Gonzalo Collantes, Georgios Petridis, Antonis Billis, Panagiotis D. Bamidis, on behalf of the LifeChamps consortium

**Affiliations:** 1 School of Medicine, Dentistry & Nursing, University of Glasgow, Glasgow, UK; 2 Academic Primary Health Care Center, Stockholm Health Care Services, Region Stockholm, 104 31 Stockholm, Sweden; 3 Research Group in Joint Research Unit in ICT Applied to the Reengineering of Socio-Sanitary Processes, Hospital La Fe de Valencia, Instituto de Investigación Sanitaria La Fe, Valencia, Spain; 4 Division of Family Medicine and Primary Care, Department of Neurobiology, Care Sciences and Society, Karolinska Institutet, 141 83 Stockholm, Sweden; 5 Lab of Medical Physics and Digital Innovation, School of Medicine, Aristotle University of Thessaloniki, Thessaloniki, Greece

**Keywords:** Older adults, Supportive cancer care, Co-creation, Digital health, Quality of life, LifeChamps

## Abstract

**Purpose:**

This study is to evaluate healthcare needs, preferences, and expectations in supportive cancer care as perceived by cancer survivors, family caregivers, and healthcare professionals.

**Methods:**

Key stakeholders consisted of cancer survivors diagnosed with breast cancer, prostate cancer, or melanoma; adult family caregivers; and healthcare professionals involved in oncology. Recruitment was via several routes, and data were collected via either online surveys or telephone interviews in Greece, Spain, Sweden, and the UK. Framework analysis was applied to the dataset.

**Results:**

One hundred and fifty-five stakeholders participated: 70 cancer survivors, 23 family caregivers, and 62 healthcare professionals (13 clinical roles). Cancer survivors and family caregivers’ needs included information and support on practical/daily living, as frustration was apparent with the lack of follow-up services. Healthcare professionals agreed on a multidisciplinary health service with a “focus on the patient” and availability closer to home. Most healthcare professionals acknowledged that patient-reported outcomes may provide “better individualised care”. Cancer survivors and family caregivers generally felt that the digital platform would be useful for timely personalised support and aided communication. Healthcare professionals were supportive of the “proactive” functionality of the platform and the expected advantages. Anticipated challenges were integration obstacles such as workload/infrastructure and training/support in using the new technology.

**Conclusions:**

Obtaining key stakeholders’ insights provided a foundation for action to further co-create the LifeChamps digital platform to meet needs and priorities and deliver enhanced supportive care to “older” cancer survivors.

**Implications for cancer survivors:**

Co-creation provided insight into gaps where digital support may enhance health and well-being.

**Supplementary Information:**

The online version contains supplementary material available at 10.1007/s11764-022-01289-7.

## Introduction

Across Europe, life expectancy has increased. Currently, on average men can expect to live till the age of 75 years and women until the age of 82 years [1]. As life expectancy increases, the risk of age-related chronic conditions such as cancer and associated factors such as frailty, comorbidities, and polypharmacy will strain healthcare services [2]. Incidence rates of cancer are strongly related to age [3], and this may bring additional challenges to delivering complex care and providing quality support for older patients who survive cancer [4]. The World Health Organisation states that there is “no typical older person” and predicts an increase of people aged 60 years and above doubling by 2050 [5]. The term “older patient” is difficult to define, as pragmatically the chronological and biological age of people differ, which can be explained by age-related physiological, cognitive, social [6], and epigenetic changes (e.g. nutrition) [7]. Also, self-perception of ageing may vary across countries and cultures [8], influencing both physical and mental health of those affected by cancer [9]. The identification of potential health issues of adult cancer survivors [10, 11] has been recommended in the cancer care setting [12], yet there remain limited resources and services to implement this in clinical practice [13].

COVID-19 has emphasised how unprepared global healthcare systems are for ageing cancer survivors [14], which may present further barriers or delayed access due to age-based (chronological age) cancer screening criteria [15]. Adult cancer survivors may often be under- or over-treated based on assumptions of functional (and/or biological) status or due to safety concerns [16]. Most of what is known is extrapolated from trials involving younger patients [17] or older cancer survivors with fewer comorbid conditions leading to further health inequalities which may impact on survivorship [16]. Adult cancer survivors with multimorbidity may find self-management difficult post-treatment due lack of support, the burden of additional tasks such as symptom monitoring, taking medications, making lifestyle changes, as well as other challenging health conditions affecting daily activities and functional abilities [18], thus increasing their risk of dying from causes other than cancer [19].

COVID-19 has compelled healthcare services to quickly adapt to technology-based solutions to manage clinical caseloads and non-emergency care [20–22]. The use of digital technology may also provide long-term solutions to overcome the challenges in resource-constrained services for cancer survivors [23]. Exploring new methods for the delivery of cancer care may offer enhanced support post-treatment as more older patients have become amenable to using telehealth services such as eHealth (e.g. internet/computer) [24], mHealth (e.g. smartphone applications) [25, 26], and telemonitoring (sensors/wearables) [27], to aid remote monitoring, health management and independent living [28, 29]. For people affected by cancer, the benefits of digital health interventions are associated with improved health-related quality of life (HRQoL) [30] and better coordination of information provision around coping strategies and health education [31]. Further evidence is needed into mHealth interventions that involve adult cancer survivors and promote engagement of physical activity, health information, weight management, and social support [30].

Older adults (aged over 50 years) have reported barriers in using telehealth such as a lack of technical literacy, desire to use, technical support, and personalisation [29]. Risks concerning privacy, security, and performance have also been reported [32]. Healthcare professionals have similar uncertainties concerning mHealth due to their lack of involvement in the development of supportive technology, accuracy of information, and the changing needs of patients with cancer [30]. Furthermore, family caregivers have expressed dissatisfaction with support and information [33], which may subsequently affect their own health [34], and less confidence when evaluating online cancer information [35]. To date, most studies have been conducted in the USA and Canada and may not be reflective of other countries, cultures, or specifically “older” adult cancer survivors [30, 35]. In the rapidly growing field of digital health, co-creation practices that integrate and link “stakeholders” in the healthcare setting are integral to shape interventions that may be beneficial to health outcomes [36]. To our knowledge, none of the digital care platforms already developed for use in cancer care are specific to “older” adult cancer survivors [37].

LifeChamps is a multinational Horizon 2020 project involving 14 partners in healthcare, academia, and industry [38]. The overall LifeChamps project aims to develop an innovative, digital platform to enhance supportive cancer care for “older” adult cancer survivors (aged 65 years or more) who require ongoing assistance. This will be directed by the use of artificial intelligence and big data analytics to integrate the following: (a) cancer and geriatric patient-reported outcomes (PROs) via mHealth [19, 39, 40]; (b) telemonitoring via home sensors and wearable devices; (c) routine clinical data from electronic health records; and (d) a web dashboard for healthcare professionals. This study reports preliminary findings from an early co-creation stage with key stakeholders (i.e. the anticipated stakeholders of LifeChamps) as we explored their perspectives on healthcare needs, preferences, and expectations to guide the design of the developing LifeChamps digital support platform.

## Methods

This study was informed by the guidance set out by the Standards for Reporting Qualitative Research [41].

### Study design

A descriptive, cross-sectional, multi-methods study was conducted. A recruitment target of 120–400 participants was planned [42] across three stakeholder groups involving four LifeChamps partner sites to obtain information-rich data. Participants were given two options to participate: either an online survey or a telephone interview. Ethics approvals were obtained separately in each country: University of Glasgow (UofG, UK); Instituto de Investigación Sanitaria La Fe (HULAFE, Spain); Academic Primary Health Care Centre of Region Stockholm (APC, Sweden); and Aristotle University of Thessaloniki (AUTH, Greece). All research activities were in accordance with the World Medical Association Declaration of Helsinki [43].

### Eligibility and recruitment

Eligible cancer survivors were diagnosed with treatable breast cancer, prostate cancer, or melanoma irrespective of diagnostic or clinical stage and were aged 50 years or above to accommodate possible differences across countries and cultures, whereby some people may consider themselves “older” earlier (aged 50–64 years) rather than later (aged 65 +). Eligible family caregivers were adults (≥ 18 years) who supported a cancer survivor aged 50 + years. Eligible healthcare professionals were multidisciplinary and involved in geriatric cancer care. All participants were able to speak, write, and communicate in their respective native language and had access to a telephone and/or email/internet.

Recruitment of cancer survivors and family caregivers at each site was facilitated by the European Cancer Patient Coalition, social media networks, patient associations, print media, and personnel employed at charitable organisations and hospitals. Healthcare professionals were recruited through professional and social media networks. All participants were free to withdraw at any point without justifying their decision.

### Procedures and data collection

Two online surveys with similar content were created, one for patients and family caregivers and another for healthcare professionals. The surveys were created by GK, developed by RMM, EK, and AG and approved by partner sites. Potential participants contacted the researchers, at the relevant sites, if interested in an online survey and were emailed the study link, which used the established EU Survey tool [44], as it provided maximum data protection, confidentiality, and translation support into multiple languages. This tool supported an embedded eligibility screener, the participant information sheet, and a consent form that required mandatory completion (e.g. agreeance to participation and their anonymous data published). Participants were provided access to the survey once they completed the consent form. Surveys were checked manually to prevent duplicates. If a potential participant did not want to proceed, they were thanked and prompted to close their internet page. If they continued, they were asked to respond to questions and view a schematic of the developing platform (referred to as “system” for enhanced clarity) (Online Resource 1). Once completed, the survey data were downloaded to anonymised password-protected files at each site.

Potential participants interested in the telephone interview were signposted to contact the researcher, who then provided them with a participant information sheet and consent form. If agreeable, all eligible participants were similarly required to complete and return a consent form, via secure email transfer. Once received, the schematic of the developing platform was emailed and arrangements made for the interview. A semi-structured telephone interview guide was prepared to reflect questions included in the surveys, which enabled rich systematic exploration of participants’ views and experiences [45]. The interviews were recorded on a digital voice recorder (e.g. Olympus VN-521PC) and transcribed verbatim.

Participants were asked 15–30 open/closed questions with the number of questions varied according to participants’ role. This also included demographic information and the Charlson Comorbidity Index [46] to identify conditions that may have a burden on participants. Duration was dependent upon the method of data collection, 15–25 min for the online survey and 30–60 min for the telephone interview. Surveys and interviews ran in parallel at the four sites. Three sites (AUTH, APC, and HULAFE) did not offer to compensate participants for their time for completing the survey (not common policy). The UofG provided participants with entry into a “lottery” for a cash prize at the end of the study, this was provided to five participants (£20 each).

### Data analysis

Descriptive statistics were used to present the demographic data and the Charlson comorbidity index. Framework analysis was used as it supports research collected at several sites, and involvement of multi-disciplinary professionals with varied experience of qualitative analysis [47]. We applied a six-step method to support data preparation and analysis, using a deductive approach [47], and this was informed by previous work [48], and upon the aim of our research question. This included (i) transcription (interviews), (ii) familiarisation with survey/interview content, (iii) coding, (iv) application of the analytical framework, (v) charting data on to the matrix using Microsoft Excel, and (vi) data interpretation. Three researchers (GK, RMM, EK) developed the working analytical framework, and final changes were made and approved by partner sites, thus creating a final version and instructions for completion. A summary of findings was completed by each site (RMM, AG, EK, PP, NP, GP) and returned to RMM and GK for final interpretation, discussion, and selection of excerpts that best reflected stakeholders’ opinions across and within sites.

## Results

### Participants’ demographics

A total of 155 stakeholders were recruited between July and November 2020, comprising 70 cancer survivors, 23 family caregivers, and 62 healthcare professionals. One hundred thirty-seven participants opted for the online survey, and 18 participants chose the telephone interview. Most cancer survivors were diagnosed with breast cancer (*n* = 60, 65%), followed by prostate cancer (*n* = 24, 26%), and melanoma (*n* = 9, 10%). Two out of three cancer survivors were in the 50–64 age group (*n* = 47, 67%) and reported fewer significant comorbidities than those aged 65 + years, as identified on the Charlson comorbidity index (Table 1). Two octogenarian cancer survivors participated (3%), whilst five family caregivers (22%) reported providing support to patients in their eighties. Family caregivers were on average younger (mean 50.3 years) than patient participants (Table 1). More than half of patients (*n* = 38, 54.3%), and those being supported by family caregivers (*n* = 10, 43.5%), had finished initial cancer treatment at least 25 months prior to participation. Overall, 61 (65.6%) participants reported a total of 38 comorbidities. Nineteen participants (31%) felt that they had got worse since finishing initial cancer treatment; this included one caregiver. The top four comorbidities reported were similar amongst patients and family caregivers, including hypertension, diabetes, osteoporosis, and heart disease.

**Table 1:** Demographics of cancer survivors and family caregivers

Variables	Responses	Cancer survivor *n* = 70	Family caregivers *n* = 23	Cancer survivors supported by family caregivers
Type of cancer (*n*, %)	Breast Prostate Melanoma	47 (67.7) 16 (22.9) 7 (10)	N/A	13 (56.5) 8 (34.8) 2 (8.7)
Gender (*n*, %)	Female Male	49 (70) 21 (30)	19 (82.6) 4 (17.4)	
Family caregiver relationship to cancer survivor (*n*, %)	Daughter Wife/partner Husband/partner Other Sister-in-law Son		10 (43.5) 5 (21.7) 3 (13) 3 (13) 1 (4.3) 1 (4.3)	
Time since end of treatment in months (*n*, %)	25 months or more 13 – 24 months Less than 1 month – 12 months	38 (54.3) 11 (15.8) 21 (30)	N/A	10 (43.5) 3 (13) 10 (43.5)
Age-group (*n*, %)	65 years and above 50–64 years Unknown (no age provided)	22 (31.4) 47 (67.1*)* 1 (1.4)	1 (4.4)	10 (43.5) 5 (21.7) 8 (34.8)
Age (mean, SD)	65 years and above 50–64 years	71.5 years, 5.2 57.2 years, 4.0	*50.3 years, 14.8	75.6 years, 8.5 61 years, 4.2
Charlson comorbidity index (median, min–max)	50–64 years + 65 years	3, 3–8 5, 3–10	1, 0–4 ^+^	5, 4–7 4, 3–8 3, 3–5
Comorbidity occurrence relative to the cancer trajectory (*n*, %)	Before cancer diagnosis After cancer diagnosis No information Side-effect of cancer treatment During cancer treatment	26 (65) 11 (27.5) 9 (22.5) 7 (17.5) 4 (40)	5 (19.2) 2 (7.7) 17 (65.3) N/A 2 (7.7)	15 (53.6) 4 (14.3) 5 (17.9) Unknown 4 (14.3)
Current status of comorbidities (*n*, %)	Stayed the same Got worse Got better No information	26 (65) 14 (35) 3 (7.5) 14 (35)	6 (23.1) 1 (3.9) 3 (11.5) 16 (61.5)	13 (46.4) 4 (14.3) 3 (10.7) 8 (28.6)

^*^Family caregivers age range, 24–76 years. ^+^Charlson comorbidity index represented whole family caregiver sample

Participating healthcare professionals represented 13 clinical roles. Most healthcare professionals were general practitioners (GP) (*n* = 14, 22.6%) followed by clinical nurse specialists (*n* = 11, 17.7%) (Online Resource 2). Six healthcare professionals were health managers. Most healthcare professionals had 11 + years oncology experience (*n* = 33, 53.1%). The most prevalent specialist area was prostate cancer (*n* = 16, 25.8%), closely followed by general medicine/practice (all cancers) (*n* = 14, 22.5%) and breast cancer (*n* = 13, 20.9%).

### Perceived healthcare needs, preferences, and expectations

We developed *10 shared themes* which provided stakeholders’ perspectives on survivorship support across our target cancers, and the requirements for the developing LifeChamps digital support platform. Representative quotes per participant group are presented in Tables 2, 3 and 4. A full account of quotes can be found in Online Resources 3, 4, and 5.

**Table 2:** Cancer survivors’ perspectives on survivorship support and the developing LifeChamps digital platform

Shared Themes	Cancer survivor themes	Coded category	Illustrative quote
(1) Stakeholders’ priorities	Priorities in life post- treatment	Finding a “new normal”	“Life since cancer has totally changed, will never be able to return to previous activities. Feel 83 not 53.” (UofG23 – aged 53 years)
(2) Stakeholders’ health concerns/needs relating to age	Health concerns/needs relating to age in survivorship	Physical/symptom Psychological/emotional	“I am worried about whether the side effects of the hormone therapy injection (back pain, stiffness, fatigue, depression) will continue. I am no longer so efficient in my work, due to constant fatigue” (AUTHGR1014 – aged 58 years) “I have also suffered from continuous depression since I was diagnosed with cancer” (HULAFESP7 – aged 82 years)
(3) Stakeholders’ experiences of support or information provision	Support/information since the end of treatment	Psychological/emotional	“Information at the right time and stop people googling for information, which may not have been proven. There is lots of false information there” (UofG27 – aged 74 years)
(4) Stakeholders’ views on family support during survivorship	Family support needs	Family-related concerns	“My husband is having cancer treatment in the same cancer hospital, which I was treated in, so I can’t escape cancer at the moment” (UofG68 – aged 63 years)
(5) Stakeholders’ concerns due to COVID	Concerns due to COVID	Practical treatment concerns	“Delays in scheduled exams” (AUTHGR1011 – aged 64 years)
(6) Stakeholders’ views on ideal health/support in survivorship	Ideal health services	Hospital/primary care/other	“A nurse led skin/ lymph gland check clinic, 6 mthly?” (APCUKP1 – aged 77 years)
	Ideal support	Practical/psychological/self-management	“How to relax and cope with worries especially those that destroy sleep” (APCUKP1 – aged 77 years)
(7) Stakeholders’ perspectives and expectations of the LifeChamps digital platform	Perceptions on the LifeChamps platform	Positive/advantages	“In principle it sounds good…advantage that someone can whenever they will to log in and get advice and help. Very positive” (APCSW4 – aged 54 years) “The sort of support you are considering would be much more applicable after my secondary cancer diagnosis, given that there is no cure” (UofGP52 – aged 67 years)
		Critique/disadvantages	“Where are the data saved? Who owns the data? Can they be sold?” (APCSW1 – aged 50 years) “person feels like a person and not an identifier for a computer” (APCUK1 – aged 77 years*)*
(9a) Stakeholders’ expectations of health professionals’ actions when using the LifeChamps digital platform	Expectations of health professionals’ actions	Communication Share information with other treating healthcare professionals	“Actually find time to read them, and talk to me as to what concerns me and will help me” (UofGP24 – aged 81 years) “the various medical specialties to collaborate with each other and exchange their knowledge and experiences, listen to the other specialty. Doctors should "listen" to both physiotherapists and nurses” (AUTHP1014 – aged 58 years)
(10) Stakeholders’ comfort with the technology suggested for the LifeChamps digital platform	Comfort with technology	Barriers reported	“I am not comfortable. I only know how to use my mobile phone to make calls (my daughter is helping me with this survey)” (HULAFEP7 – aged 82 years)

Theme 8, frequency of predictions, refer to illustrative online resource 3

**Table 3:** Family caregivers’ (CG) perspectives on survivorship support, and the developing LifeChamps digital platform

Shared theme	Family caregivers theme	Coded category	Illustrative quote
(1) Stakeholders’ priorities	Family caregivers’ own priorities since supporting the survivor	Family life Psychological/emotional	“I continue with the same life that I had during the treatment, as she is an older person I have to continue with her care” (HULAFESP11 – CG aged 58 years, patient aged 82 years) “Somebody to talk through my experience with so I felt less alone” (UofG2 – CG aged 30 years, patient aged 64 years)
(2) Stakeholders’ health concerns/needs relating to age	Health needs/concerns relating to age of the survivor	Psychological/emotional Physical/symptom	“She is afraid of cancer recurrence” (HULAFESP3 – CG aged 55 years, patient aged 82 years) “To survive” (AUTHGR1017 – CG aged 24 years, patient aged 82 years)
(3) Stakeholders’ experiences of support or information provision	Survivors needs for support	Psychological/emotional	“This is a mental health issue, and another one that’s very rarely talked about, the mental health to help him, to allow other people to help him with this would be good” (UofG34 – CG aged 62 years, patient aged 64 years metastatic cancer)
(4) Stakeholders’ views on family support during survivorship	Family caregivers’ need for support Cancer survivor support	Psychological/emotional	“Somebody to talk through my experience with so I felt less alone” (UofG2 – CG aged 30 years, patient aged 64 years) “Definitely psychological to accept the change he is experiencing and the limitations he now has in his life” (AUTHGR1016 – CG aged 25 years, patient age unknown)
(5) Stakeholders’ concerns due to Covid-19	Caregiver and survivor concerns due to Covid-19	Psychological/emotional	“I am concerned about the lack of healthcare for any disease other than coronavirus” (HULAFESP12 – CG aged 55 years, patient aged 82 years)
(7) Stakeholders’ perspectives and expectations of the LifeChamps digital platform	The developing LifeChamps platform	Positive/advantages Critique/disadvantages	“Continuous psychological and physical support of both the patient and those involved in such a condition is important. So, we need a system that constantly supports the citizens” (AUTH1016 – CG aged 25, patient age unknown) “For older people it would be better if the system worked through caregivers” (HULAFESP11 – CG aged 58 years, patient aged 82 years)
(9a) Stakeholders’ expectations of health professionals actions when using the LifeChamps digital platform	Expectations of healthcare professional’s actions	Communication Adjust follow-up care	“A deep understanding of what is going on in the patient’s mind and body. Emphasis on parity and the inclusive open procedure between doctor and patient” (AUTHGR1023 – CG aged 50 years, patient aged 87 years) “To speak with the patient to advise treatment and a way of life.” (HULAFESP12 – CG aged 55 years, patient aged 82 years)
(10) Stakeholders’ comfort with the technology suggested for the LifeChamps digital platform	Comfort with technology	Positive/advantages	“I use my phone for everything, but it used to be computers for everything” (UofGC34 – CG aged 62 years, patient aged 64 years)

Themes 6 and 8, refer to illustrative online resource 4

**Table 4:** Healthcare professionals’ perspectives on supporting cancer survivors, and on the developing LifeChamps digital platform

Shared theme	Healthcare professional theme	Coded category	Illustrative quote
(1) Stakeholders’ priorities for cancer survivorship	Healthcare professionals’ priorities for support	Best supportive care	“Priorities are driven from individual's limitations in function-structure, activities-participation” (APCSW11) “To empower them to return to functional meaningful day to day life if their state of health allows that” (UofGHP34)
(2) Stakeholders’ health concerns relating to age	Perspectives of cancer survivors’ post-treatment health needs	Physical symptoms Psychological support	“Support to help regain fitness or energy after treatment” (UofGHP3) “Disfiguration is a continuing reminder of the undergone illness” (APCSW6)
(3) Stakeholders’ experiences of support or information provision	Views on age-related support for patient/family post treatment	Practical/daily living Psychological support	“People always seem to struggle with managing fatigue and want information” (UofGHP12) “They have a lot of questions, they have information gaps for the continuation of their monitoring too, especially the older people” (AUTHGR0008) “To listen and show understanding” (APCSW6)
(5) Stakeholders concerns due to COVID-19	Views on cancer survivors’ concerns due to COVID-19	Access to health services Daily living and community care	“Extra-long waiting time when visit postponed because of infection symptoms of the patient or clinician” (APCSW12) “Increased deconditioning and significant loss of muscle strength and exercise tolerance, lack of confidence, increased anxiety, increased fatigue, significant psychological impact of shielding” (UofGHM10)
(6) Stakeholders’ views on ideal health services and support in survivorship	Views on health services post-treatment	Primary care Remote monitoring Hospital follow-up	“Primary health care near their place of residence, instead of hospital care and treatment” (AUTHGR1025) “Contact nurse with feasibility to flexible telephone hours” (APCSW12) “Nurses, primary care doctors, geriatrician in the case of older patients, physiotherapists, psychologists and the specialist in charge of their cancer” (HULAFESP2)
(7) Stakeholders’ perspectives and expectations of the LifeChamps digital platform	Perspectives on the LifeChamps system	Praise/advantages Critique	“The great advantage is that it allows quantifying things that seem intangible and can allow early intervention” (HULAFEHP3) “Every patient is different. You can hardly categorize them. We need almost as many categories as patients.” (AUTHHP1037)
	Expectations of health professionals/required information and monitoring	Practical and daily living	“Information that would be beneficial include changes in function and QoL, awareness of who is (or isn't) utilising self-management advice and implementing behavioural changes relating to preventative strategies.” (UofGHM10)
	Requirements for implementation	Tackle workload/ human resource barriers	“As everywhere, workloads are stretched—if this system is burdensome, it unfortunately will not be well used. Main thing would be ease of use, support, info that is beneficial to patient assessment” (UofGHP30)

#### (1) Stakeholders’ priorities for cancer survivorship

Across sites, cancer survivors most frequently identified “finding a new normal” as their priority in life as the consequences of cancer had a varying degree of impact on patients’ lives. Most family caregivers felt that they had returned to normal and were enjoying and appreciating family life, but for some, their caring responsibilities remained despite their family member finishing cancer treatment, and they were still feeling emotional. Overwhelmingly, healthcare professionals’ priority was to provide the best supportive care, which varied according to the patient’s diagnosis, their individual priorities, and the support structure at each site. Frequent monitoring was described (Greece), with the emphasis on survival (Spain). Across all sites, there was little reference to the importance of communication with cancer survivors during survivorship. One healthcare professional did acknowledge that “often patients do not take in or remember all the information they are given at hospital” (UK).

#### (2) Stakeholders’ health concerns/needs relating to age

Cancer survivors both below and above the age of 65 had two main areas of unmet needs: symptom-related and psychological/emotional. Similar shared concerns across the age spectrum included fear of cancer recurrence, medication side-effects, and bone health (“aching bones,” “bone density”). However, the need for psychological support was more evident in the 50–64-year-old group, suggesting possible interactions between unmet needs such as “poor sleep”, “pain/stiffness”, “stress”, and “anxiety”. This may be especially important for those patients who still need employment, which was identified in the theme *ideal support.*

Family caregivers, irrespective of cancer survivor age group, described similar areas of concern in relation to physical or symptom-related and psychological/emotional needs. Family caregivers provided insight into their experiences of supporting patients who were older, in that they may need a “little more help” but did “not want to become a burden”.

Healthcare professionals viewed the management of the patient’s physical symptoms as the main health need post-treatment, across the age spectrum, and specific issues described were “fatigue”, “pain”, and “loss of fitness”. Psychological support was also viewed as important, with concerns over the fear of cancer relapse, mood changes, and increased sleep disturbance, as similarly described by patients’ themselves.

#### (3) Stakeholders’ experiences of support and information provision

Cancer survivors identified that psychological/emotional support and the need for information were the two most important areas of concern between finishing treatment. Cancer survivors wanted information available to them at the right time and from factual and reliable sources that covered a range of concerns such as healthy eating and mental well-being. The lack of clear communication and direction of where to go for help was described as “fear” or “worry” and reluctance in contacting their GP. Cancer survivors expressed more information and psychological support was available through sharing experiences with peers and patient support groups, than what was received via their healthcare professionals.

Most family caregivers identified that they needed psychological/emotional support themselves or for their partner, especially if the patient they were caring for had non-curative cancer. Furthermore, family caregivers who were being kept informed about their patient’s treatment and health could feel some sense of control as they had better awareness of their symptoms, “I was informed about the treatment he is receiving and the state of his health due to diabetes in combination with cancer” (Greece).

Healthcare professionals across all sites identified the need for support and information in key areas of practical and day-to-day living such as managing fatigue, increasing physical activity, and psychological support. They also highlighted the importance of time to provide “information” and to explain “adverse effects that the patient may not be aware of because they are not treatment related” (Spain). Gaps were also identified in the continuation of the monitoring progress, especially in older adults (Greece).

#### (4) Stakeholders’ views on family support during survivorship

There were very few cancer survivors who identified support was needed for their families across sites. This may be due to most cancer survivors completing their treatment 25 months earlier to their involvement in this survey, or due to individual circumstances as some patients lived alone. It was suggested that there was need for support to help with adjustment, especially as there maybe more than one person in the family going through cancer treatment.

Family caregivers had identified they needed psychological/emotional support themselves, or for their partner, especially if that person had non-curative cancer. This also resonated in their perspectives of the current support needed as family-patients were having issues with treatment-related side effects and acceptance of life changes. Across the four sites, providing family support did not emerge as a theme during data collection analysis for healthcare professionals, which may highlight the general lack of support to family caregivers.

#### (5) Stakeholders’ concerns due to COVID-19

Cancer survivors described the practical aspects of treatment as their most common concern. This may reflect how the pandemic has affected some patients more than others, perhaps depending on their diagnosis/point of cancer survivorship and the rate of infection across countries.

Overall, the results revealed that family caregivers experienced social unease and were being “precautionary”, and there was also concern for loss of other health services. Most family caregivers viewed their patients as requiring emotional support and depicted them as being “frightened”, “depressed”, or feeling “vulnerable” due to myelosuppression, but also the physical/symptoms as family-patients were concerned about “getting sick” from the virus.

Most healthcare professionals described that those older patients were experiencing concerns with access to health services, and this contributed to “anxiety” and “delays” with appointments, and “*fear of visiting hospitals*.” This was closely followed by problems with daily living and community care as some patients reported psychological effects such as “feeling lonely” and “isolated”. Issues with follow-up services were more pronounced in some countries as “non-compliance” to appointments and visits were described (Greece).

#### (6) Stakeholders’ views on ideal health services and support in survivorship

There was a varied response to what support was experienced and offered in existing health services across countries. Patients felt that physiotherapy, physical activity, and psychological support were most needed. Although the service responsible differed (i.e. hospital, primary care), there was agreeance that it should be provided free of charge. Patients described “other” health services such as those that offer specific support in areas such as a nurse-led skin clinic (Sweden), diet (UK, Spain), and counselling (Greece, UK). These types of services could be interpreted as “holistic”, or rather a service “where post-cancer patients are fully monitored” (UK). Furthermore, cancer survivors across the age spectrum commonly identified areas of need in practical day to day living, psychological support, and self-management. Their expectations were to have information/advice that reflected realistic concerns regarding how to prepare for life post treatment such as what are “normal feelings”, how to cope with returning to work (50–64 years of age), and type of diet. Self-management reflected the need for support with relaxation and to give them a sense of control over side effects of treatment.

An overall interpretation was collated from family caregivers’ perspectives as few provided their views on an ideal health service or type of advice/information which would benefit patient’s post-treatment. Family caregivers appeared to be unclear of where to get support, “Who is now there to help you on how to access services easily” (refer to online resource 4).

Healthcare professionals’ mainly provided insight into primary care services, which may reflect the number of GP’s involved in this study, and the need for improvement of the services offered, especially in the community. Services in the community may be more of an issue for those requiring home help (Greece). Hospital follow-up services described a need for a specialised onco-geriatric service involving a multi-disciplinary team (Spain). Flexible and remote monitoring with a contact oncology nurse was seen as a priority for cancer survivors with melanoma (Sweden). Overall, there was agreeance that a health service which combined of a range of specialities that could “collaborate” and “focus on the patient” closer to home would be beneficial to older cancer survivors.

#### (7) Stakeholders’ perspectives and expectations of the LifeChamps digital platform

Most cancer survivors’ views were positive, especially for easy access and timely information when they needed support. One perception encapsulated the general opinion with, “whatever it is, must be detailed, scientifically accurate and not patronising” (UK). Patients regarded the platform as a good opportunity to help the healthcare system work better by filling gaps in the current delivery of care, either through better prevention or by helping with follow-up care. Patients diagnosed with secondary breast or prostate cancer stated that the type of support being developed would be more applicable after secondary diagnosis. There was some critique on the platform, but this was mainly on how information would be presented and what would happen to their data.

Family caregivers perceived the development of the platform as advantageous as it would be able to offer constant support to cancer survivors and might suit older people if operated through the caregiver. Of note, was the opinion that the platform may “predict issues that may not occur”, which may increase the risk of unnecessary heightened anxiety.

Overall, feedback from healthcare professionals was positive and supportive towards the “proactive” effects the proposed technology could have on monitoring older cancer survivors’ health status. Healthcare professionals’ felt that the platform could help identify, specify, and quantify cancer survivors’ needs that are currently not being taken into account, flag cancer survivors at risk for declined health status, and do so in an objective and tangible way. Although there was almost as much critique on how the platform could be managed, safely accessed, or communicated within the existing healthcare system infrastructure, impact on workload and issues around patient safety such as frequency and timely actioning of patient feedback was also mentioned, which are essential points to be considered when deploying the platform in clinical practice. Also, it was emphasised that accessibility to the platform should be an important aspect for consideration, from a patient point of view, especially as the platform is intended to support “older” cancer survivors.

In relation to the type of information required from the platform, healthcare professionals would like monitoring/management of specific symptoms such as endocrine treatment side-effects, activity at night, exercise and nutrition, and patient functioning. Other healthcare professionals hoped that the platform would help monitor (a) physical indicators such as “accidental falls, weight, physical activity”; (b) indicators of cancer “suspected recurrence”; (c) mental health status, specifically depression; and (d) adherence to medical advice or prescribed medications “degree of completion of the recommendations given”.

Regarding the presentation of information, a summary report was suggested as useful to provide healthcare professionals with details necessary to evaluate each patient case individually and over time. Healthcare professionals involved in this study foresee that a collaboration would be necessary between the different stakeholders (e.g. healthcare professionals’, cancer survivors), on top of investment in time and technology. However, many stressed the necessity for compatibility of this information with the electronic health record for the platform to be integrated into routine cancer care (e.g. through mobile applications, the medical record system or online). Healthcare professionals also emphasised the need for clear pathways about who acts upon the information from the platform, the need for adequate IT support in the everyday use of the platform, user-friendliness, accessibility, and involvement of the wider multidisciplinary team to tackle known workload and human resources barriers. Automation and technological compatibility were cited as aspects to consider in order to tackle infrastructure barriers, whereas adequate time for training of the involved staff could facilitate a buy-in process.

#### (8) Stakeholders’ views of the frequency of receiving summaries/predictions/advice from the LifeChamps digital platform

Cancer survivors’ views were not very specific, and suggestions varied between every 3 months, 6 months, or annual summaries/predictions depending on the needs of the person, which on reflection could also have been coded as “on demand.” Patients had no specific preference to receiving advice regarding their health, but it was clear that cancer survivors felt this was very much dependent “on the need” for support (refer to online resource 3).

Family caregivers considered having a regular update on the patient’s situation as acceptable. Similar to cancer survivors, a wide variability in responses was noted, which pointed to the direction of regular updates provided at least a couple of months apart (if not longer) (refer to online resource 4). Most healthcare professionals felt that “on-demand” information before every patient visit, or approximately every 3–4 months, would be reasonable to provide an update on a patient’s health status (refer to online resource 5).

#### (9) (a) Stakeholders’ expectations of health professional actions when using the LifeChamps digital platform

Cancer survivors clearly wanted to see improved communication between themselves and healthcare professionals in response to the developing platform. As evident throughout the data collected, there was a sense of frustration with the lack of post-treatment services. Cancer survivors would like healthcare professionals to use the platform to discuss their data with them and tailor clinical decisions to their own needs. There was an expectation that the platform would facilitate better collaboration and communication between different healthcare professionals involved in the patient’s care.

Family caregivers expected the platform to steer healthcare professionals towards setting expected patient goals. Simultaneously, this could provide healthcare professionals with a realistic insight and a better understanding of the physical and mental health of their cancer survivors. For development purposes, this would involve a range of proactive actions to enable adjustment of care, whereby healthcare professionals would alert cancer survivors and family members if they detected a problem, as well as provide advice about possible future issues.

#### (9) (b) Healthcare professionals’ views on the use of patient-reported outcomes via the digital platform

Healthcare professionals’ expectations were reported earlier (theme 7), and we specifically asked their views on patient-reported outcome and measures currently used in their clinics with older cancer survivors, and/or what would be most important during survivorship. Most healthcare professionals did not comment on this question. However, those who responded were mainly supportive of the use of patient-reported outcome measures and mentioned measures used locally and internationally such as EQ-5D-5L, FACT, and the Distress Thermometer (UK and Greece) and the benefits of using patient-reported outcome measures to provide “better treatment of the individual patient” in areas such as physical activity, fatigue, diet, and quality of sleep (refer to online resource 5).

#### (10) Comfort with the technology suggested for the LifeChamps digital platform

Most cancer survivors were comfortable using the proposed technology such as a mobile app and a fitness tracker. However, some barriers were reported as cancer survivors would prefer “face-to-face” care rather than through a device. A few older cancer survivors reported that they did not know how to use a mobile phone other than for calls and that this may cause additional stress in their lives. Most family caregivers were more than comfortable with everyday use of technology. Healthcare professionals’ use a variety of platforms as part of their professional roles and had identified the need for this to be user-friendly and to have technical support available (theme 7).

## Discussion

We achieved our research aim of exploring perspectives of key stakeholders on their healthcare needs and preferences and expectations of the LifeChamps digital platform. Collectively, we surpassed the minimum recruitment target of 120 participants in total, across groups, which enabled rich data collection. To summarise our main findings, cancer survivors identified better communication as one of their healthcare needs, particularly in relation to where to go for reliable information and support following initial cancer treatment. This remained a significant issue even though most cancer survivors had completed treatment 25 months prior to participation. Fitch et al. [49] also reported that “finding the support I need” was one of the main challenges for older cancer survivors up to 3 years post-treatment in Canada. In our study, this accentuated the mixed availability of health services and support for some people still needing more assistance (e.g. how to cope). The use of PROs may improve communication between patients-clinicians before discharge for those on curative treatment. Similarly, for those with non-curative disease who continue with treatment and have longer periods between follow-ups, PROs may benefit by offering more personalised and timely early intervention. Barriers to incorporating PROs have been reported before [50], and this is one aspect being explored in the development of the LifeChamps project.

Furthermore, some healthcare professionals expressed a priority for frequent monitoring, with focus on survival (Greece and Spain), and all were trying to provide the best supportive care within their infrastructure. However, improved communication was not a conscious “priority” across all four countries and was a key frustration experienced by cancer survivors’ post-treatment. Likewise, in the USA, cancer survivors expressed frustrations with communication and obtaining information to prepare for the challenges associated with cancer survivorship and chronic care needs [51].

In relation to age, our study reported on two main areas of unmet healthcare needs across the age spectrum, physical problems, as part of their ‘new normal life’, with particular concerns in relation to bone health, and psychological/emotional difficulties which were prevalent (e.g. depression, coping), and identified by all stakeholder groups. Psychological issues continued longer than expected, more so in a 50–64-year age group. Family caregivers also felt that more psychological support was needed for both cancer survivors and often themselves. Family caregivers’ provided insight into the complexity of older cancer survivors who may not want to mention they need some help, and cancer survivors themselves being reluctant to contact a GP. Similarly, Corbet et al. [18] reported on the process of cancer survivors weighing up whether they thought an issue was troublesome enough to mention. Overall, these different factors need to be better communicated with healthcare professionals’, especially so they can provide effective support (e.g. content, timing) to cancer survivors and family caregivers to meet their evolving needs [52]. Indeed, most of these gaps were identified by healthcare professionals in our study, such as the need for information/support on practical day-to-day living for adult cancer survivors, but lack of time and existing healthcare infrastructure were an obstacle.

We found healthcare professionals scarcely used patient-reported outcome measures with adult cancer survivors across the four countries. Yet, a key finding was that all healthcare professionals who responded were agreeable that this would allow better treatment of the individual patient, and this adds further support to the incorporation of patient-reported outcome measures to provide optimal care [40]. Awareness, education, and understanding of how early intervention may maintain or improve health, well-being, and independence in an ageing population, within different clinical settings, are just as important as survival itself [19, 39].

Ideal health services identified by stakeholders varied across countries, yet one of our key highlights was the overall agreeance for a “holistic” service providing support from multi-disciplinary professionals that could focus on the patient’s needs, collaborate, and be available in the community, especially in the context of the complex needs of adult cancer survivors with cancer. More so, as we progress into a post-pandemic period with increased healthcare waiting times.

Stakeholders’ perspectives on the developing digital platform were generally positive. Cancer survivors and family caregivers felt that it would be useful for personalised support, especially if recommend by their healthcare professional, by providing easy access, timely support when they needed it, preventing “googling” which could lead to false information, and aiding communication due to the frustration with the lack of follow-up services. Similarly, recommendations were found to influence older people to sources for information, in Germany [25]. Some cancer survivors felt that the digital platform would be more applicable to them after secondary diagnosis due to the changes experienced. Our study included perspectives from individuals who had metastatic cancer, or were caring for a family member, and are often underrepresented in research [53]. Most of the criticisms received were on how information was to be presented and who would have access to their data, which was similar to previous research [32]. The risk that false summaries/predications may cause increased stress/anxiety was reported by one person; however, telehealth interventions (including mHealth/eHealth) have been reported to decrease psychological stress and increase autonomy and cognitive abilities in older people [29]. There were a few concerns of people aged 75 + years on comfort of using this technology, which will inform the development process [35, 54]. People aged 65 years and above are increasingly active [55], and the LifeChamps approach aims to address the specific needs of patients including digital literacy by offering training and support to those who may not have had the chance before (e.g. lower education, lack of opportunity, ageism). Adoption of this remote monitoring technology may also better support those patients who live rural by enabling better patient-clinician communication between infrequent visits and alleviating barriers to support/services due to distance. Whilst the feasibility of the LifeChamps project will be evaluated in a real-world setting using low-cost off-the-shelf devices and sensors, the preference for this mode of delivery is not one size fits all, but may enhance patient choice.

To respond to previous concerns [30], we involved healthcare professionals in this co-creation study, which revealed overall support of the functionality of the digital platform to monitor older cancer survivors’ health and activity status, HRQoL, including patient-reported outcome measures outside of real-time clinical appointments. Anticipated challenges included issues with information management, impact on workload and infrastructure barriers, and requirement of support in using technology and clinical recruitment, and by addressing some of these issues may facilitate a buy-in process from other healthcare professionals.

Overall, this study provided the perspectives from various stakeholders on the developing LifeChamps digital platform and how this information will progress future work to achieve the aims of the project (Table 5).

**Table 5:** Themes potential influence on the design development of the LifeChamps digital platform

Theme	Design development
(1) Stakeholders’ priorities	• Influence on the questions to be answered when first using the LifeChamps mobile app to enable users (participants) to personalise it to their current concerns and highest priority • Enable personalised educational content, according to identified user priorities, when using the LifeChamps mobile app • Influence on quality-of-life topics for further investigation through LifeChamps exploratory analytics algorithms
(2) Stakeholders’ health concerns/needs relating to age (3 & 4) Stakeholders’ experiences of support /information provision	• Enable users to access relevant educational components and information on sources of support on the LifeChamps mobile app • Enable personalised motivational content when using the LifeChamps mobile app • Enable continuous monitoring of physical and psychological symptoms using the LifeChamps mobile app • The design suitability of the LifeChamps mobile app will be explored further with ‘older’ adults in subsequent development cycles
(6) Stakeholders’ views on ideal health/support in survivorship	• Create coping categories for users to explore on the LifeChamps mobile app • Appropriate messages sent to encourage, support, and motivate users
(7) Stakeholders’ perspectives and expectations of the LifeChamps digital platform	• Provide clear understandable information of what is happening to participants’ data • Subsequent study recruitment materials will clarify that technology will not substitute usual health professional care for participants
(8) Stakeholders’ views of the frequency of receiving summaries/predictions/advice from the LifeChamps digital platform	• Data visualisation should be offered for all users (patients and healthcare professionals) during development cycles
(9) Stakeholders’ expectations of health professionals’ actions when using the LifeChamps digital platform	• In the developing feasibility study healthcare professionals will view participating patients’ data, via the web dashboard, and be asked their views on clinical relevance and whether this could influence patient care • Data collected could be viewed by different healthcare professionals in the feasibility study • Potential processes when patterns of health or support status change. This could be explored through advanced Artificial Intelligence analytics, e.g. when changes are not in line with quality and safety standards
(10) Stakeholders’ comfort with the technology suggested for the LifeChamps digital platform	• Provide training and support to all users, if they want it, so they are comfortable using the digital health devices

Not included theme 5 which refers to COVID-19

The main limitations of this study originated primarily from amendments to the original methodology to absorb the impact of the COVID-19 pandemic. We were unable to access usual routes of recruitment as health services and staff and charity/support groups were limited and resources stretched, which was reflected in the number of participants recruited. Our recruitment was largely reliant upon members of the public, via online/social media routes, and we found that family caregivers were difficult to engage. This may be due to several factors such as the change in our recruitment methodology; also, most of our responses were from independent adult cancer survivors (aged 50–65 years), possibly with a more favourable diagnosis requiring fewer family members to provide them with support. Whilst we recruited 22 people aged 65 years and above, there were fewer cancer survivors aged over 70 years; thus, the wider perspectives of this age group may be missing. Similarly, a small number of people participated who were diagnosed with prostate cancer or melanoma. However, the perspectives collected suggested how a digital remote platform may have enhanced the support much needed during this time. Our sample may be more biased towards survivors and caregivers who were already accessing information online, which may have resulted in missed perspectives from older cancer survivors who were not familiar with using technology. Furthermore, no data was collected to identify participants’ literacy or income deprivation which may relate to the degree of comfort or access some participants have using the proposed technology.

## Conclusion

We identified a mixture of survivorship care mechanisms as well as gaps in cancer care services across four countries. Expectations of the proposed integration of digital technology, such as mHealth, telemonitoring, electronic health records, and patient-reported outcome measures, also varied. Further engagement with “older” cancer survivors will be required. However, these findings address some of the identified health needs and enhance supportive care for adult cancer survivors (at least) with breast cancer, prostate cancer, and melanoma. Stakeholder co-creation provided insights into the digital platform requirements and provided a strong foundation to aid further development. Research into feasibility methods, cost-effectiveness, training, and support, such as remote and passive digital healthcare in the community, is warranted.

## Supplementary Information

Below is the link to the electronic supplementary material.

Supplementary file1 (PDF 141 KB)

Supplementary file2 (DOCX 17 KB)

Supplementary file3 (DOCX 21 KB)

Supplementary file4 (DOCX 21 KB)

Supplementary file5 (DOCX 38 KB)

## Data Availability

Data that support the findings of this study are included in this article and its supplementary files.
