# Supplementary material for: A multinational investigation of healthcare needs, preferences, and expectations in supportive cancer care: co-creating the LifeChamps digital platform
Source: J Cancer Surviv. 2022 Nov 11;17(4):1094–110. doi: 10.1007/s11764-022-01289-7 (PMC9650169; doi:10.1007/s11764-022-01289-7)
Supplement: Supplementary file 1 — Supplementary file1 (PDF 141 KB) [file 11764_2022_1289_MOESM1_ESM.pdf]

What are we doing this interview for?

In this project we want to develop a system that will identify those patients who need additional support after treatment for cancer. The system will use information such as the patients age, gender, physical activity or his/her quality of life to try and predict future problems with the patient's health and offer personalised advice to prevent, or manage these problems, if they occur. It will also inform the clinical team where the patient needs additional help. We have put what this may look like in the picture below.

We will ask you some questions to see what you think about a system like this.

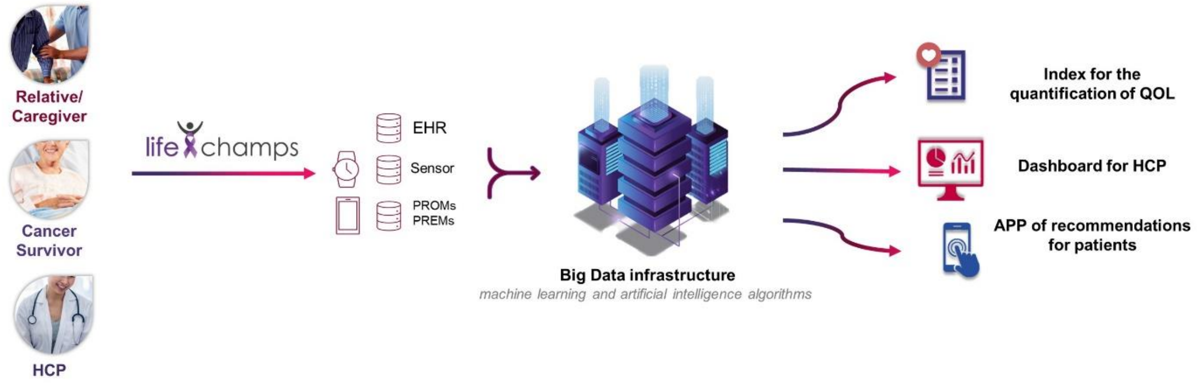

Information about the words used in the above diagram.

HCP means health care professional involved such as a doctor or nurse.

EHR means Electronic Health Records, which are already kept on computer.

Sensors are objects that might be used to monitor symptoms (e.g., a watch or mobile phone apps).

PROMs / PREMs are the questionnaires completed by the patient to tell the clinician how they have been able to achieve daily tasks (e.g., climbing stairs) or their own experiences (e.g., how they are feeling).

Big Data Infrastructure means the processing and analysis of information that will come from EHR, Sensors and PROMs / PREMs.

Quantification of QOL (quality of life) means how much has life returned to "normal".

Dashboard for HCP means the information the new device will be able to send via the electronic system (computer) to the clinician.

APP means the type of software downloaded on your mobile that may help (e.g, with management of symptoms)
