# Supplementary material for: A multinational investigation of healthcare needs, preferences, and expectations in supportive cancer care: co-creating the LifeChamps digital platform
Source: J Cancer Surviv. 2022 Nov 11;17(4):1094–110. doi: 10.1007/s11764-022-01289-7 (PMC9650169; doi:10.1007/s11764-022-01289-7)
Supplement: Supplementary file 2 — Supplementary file2 (DOCX 17 KB) [file 11764_2022_1289_MOESM2_ESM.docx]

Online Resource 2: Healthcare Professionals’ Demographics

| Variables | Responses | Health Care Professional n = 62  N (%) |
| --- | --- | --- |
| Gender | Female  Male  Prefer not to say | 44 (70.9)  17 (27.4)  1 (1.6) |
| Role | General Practitioner  Clinical Nurse Specialist  Clinical Oncologist  Urologist  Physiotherapist  Medical Oncologist  General / Community/ District Nurse  Psychologist  Plastic Surgeon  Specialist Radiographer  Occupational Therapist  Dermatologist  Dietician | 14 (22.6)  11 (17.7)  10 (16.1)  8 (12.9)  5 (8.1)  4 (6.4)  3 (4.8)  2 (3.2)  1 (1.6)  1 (1.6)  1 (1.6)  1 (1.6)  1 (1.6) |
| Time working specifically in cancer (years) | 21+  16 – 20  11 – 15  6 – 10  1 – 5  Less than 1 year  Other | 11 (17.7)  11 (17.7)  11 (17.7)  9 (14.5)  9 (14.5)  8 (12.9)  3 (4.8) |
| Time working in current role (years) | 21+  11-15  6 – 10  1 – 5 | 2 (3.2)  13 (20.9)  20 (32.2)  27 (43.5) |
| Specialist area/s* | Prostate cancer  General Medicine / Practice (all cancers)  Breast cancer  Melanoma  No information  Chemotherapy  Nursing / Palliative care / lymphoedema  Psychology  Rehabilitation  Lung cancer  Hematology (all cancers)  Hospice (all cancers)  General musculoskeletal (all cancers) | 16 (25.8)  14 (22.5)  13 (20.9)  6 (9.6)  4 (6.4)  4 (6.4)  2 (3.2)  2 (3.2)  1 (1.6)  1 (1.6)  1 (1.6)  1 (1.6)  1 (1.6) |

(*Some healthcare professionals have more than one area of specialty)
