# Supplementary material for: A multinational investigation of healthcare needs, preferences, and expectations in supportive cancer care: co-creating the LifeChamps digital platform
Source: J Cancer Surviv. 2022 Nov 11;17(4):1094–110. doi: 10.1007/s11764-022-01289-7 (PMC9650169; doi:10.1007/s11764-022-01289-7)
Supplement: Supplementary file 3 — Supplementary file3 (DOCX 21 KB) [file 11764_2022_1289_MOESM3_ESM.docx]

Online Resource 3: Illustrative quotes on cancer survivors’ perspectives on survivorship support, and the developing LifeChamps digital platform

| Shared Theme | Theme | Coded category | Illustrative quote |
| --- | --- | --- | --- |
| (1) Stakeholders’ priorities for cancer survivorship | Priorities in life post-treatment | Finding a ‘new normal’ | "It's a new normal for erectile dysfunction and a bit of urinary incontinence." *(HULAFESP5 - aged 67 years)*  "I can’t crouch down anymore, I can’t do anything like that, since I’ve had the chemotherapy, I would say that I’m even struggling with that" *(UofG28 – aged 60 years)*  “returned to normal life although with much more awareness of my health and well-being” *(APCUKP2 – aged 76 years)* |
| (2) Stakeholders’ health concerns/needs relating to age | Health concerns/needs relating to age in survivorship | Physical/symptom  Psychological/emotional | "As a result of the treatment, I lost my taste in many foods." *(HULAFESP8 – aged 78 years)*  "UK hospitals and doctors try to make you believe problems are "due to your age." *(UofG24 – aged 81 years)*  "There is very little support for the patient too once you have survived for 5 years, you are regarded as a success by NHS and get signed off all support services." *(UofG57 – aged 56 years)*  “the possibility that there might be a recurrence...mild anxiety about being in the sun” *(APCUKP3 – aged 60 years)* |
| (3) Stakeholders’ experiences of support or information provision | Experiences of support and information since the end of treatment | Information  Psychological/emotional | “I would have liked to have written information about what type of melanoma according to the scales and subtypes I had” *(APCSWP4 – aged 54 years)*  "From doctors’ little information, more information through patient associations" *(HULAFESP4 – aged 63 years)*  “Information regarding healthy eating and mental well-being” *(AUTHGR1011 – aged 64 years)*  I think there might be a fear out there amongst some men about what might…the future might hold. One of the things about cancer is you do worry about contacting your GP and discussing things" *(UofG33 - aged 66 years)*  “I needed psychotherapy for a while to accept the situation and overcome it as well as the fears I had for a possible metastasis.” *(AUTHGR1010 – aged 52)* |
| (4) Stakeholders’ views on family support during survivorship | Family support needs | Family-related concerns | "After so long I think we have quite overcome the bad times" *(HULAFESP1 – aged 61 years)*  I am alone” (APCSWP1- aged 50 years) |
| (5) Stakeholders’ concerns due to Covid-19 | Cancer survivor concerns due to Covid-19 | Practical treatment concerns | “Oncology appt moved four times since Aug. GP sent me for a CT scan as I have shortness of breath. Small area on scan they have put down to covid - it was a small blurry area which was cancer before." *(UofG50 – aged 59 years)*  “less face to face and more telephone or video consultations” *(APCUKP3 – aged 60 years)*  "I am concerned should the cancer return and treatment delayed due to the focus on covid 19 in the NHS." *(UofG26 – aged 52 years)* |
| (6) Stakeholders’ views on ideal health services and support in survivorship | Ideal health services | Hospital/Primary care/Remote/Other | “Immediate access to doctors when necessary, physiotherapy by specialists, mental health, free physical activity services (exercise should be included in health services).” *(AUTHGR1012 – aged 50 years)*  "Psychological Support" (*HULAFESP8 -* aged 78 years)  "Counselling; healthy diet (dietician); massages; Reiki" *(UofG58 – aged 57 years)* |
|  | Ideal support | Practical/Psychological/self-management | "solution-focused therapy, cognitive behavioural therapy or just somebody listening to me, saying how I feel, or I felt, at the end of it." *(UofG28 – aged 60 years))*  “Clear prognosis for the course of my health and coverage of the side effects of the treatment” *(AUTHGR1011 – aged 64 years)*  "Information about diet and possible new tumours" *(HULAFESP3 – aged 58 years)*  "Details about what support was out there in the community. Help with return to work - tiredness, forgetfulness, being overwhelmed by the noise" *(UofG30 – aged 59 years)* |
| (7) Stakeholders’ perspectives and expectations of the LifeChamps digital platform | Cancer survivor perspectives of the LifeChamps platform | Positive / advantages | "I think with an app you can go onto it any time you feel actually, I need a wee bit of support here. I think an app would be quite good, actually…. "But if somebody would just tell me that or somebody would suggest it to me from the NHS" *(UofGP28 – aged 60 years)*  "I wonder is there still a concern that people of my age, men of my age, how they respond to the electronic message but there will be people within a family that do respond to the electronic message. I think we are more prepared for hearing about or getting information about coping strategies” *(UofGP33 – aged 66 years)*  “I consider the personalised counselling as an advantage as it will offer a better quality of life to the patient” *(AUTHP1012 – aged 50 years)*  "I see it quite useful especially for prevention" *(HULAFESP1 – aged 61 years)*  "It would be an ideal system for the follow-up of cancer patients." *(HULAFESP3 – aged 58 years)* |
|  |  | Critique / disadvantages | “Where are the data saved? Who owns the data? Can they be sold? A disadvantage can be that I do not feel I am getting something new and it will just take space in my phone.” *(APCSW1 – aged 50 years)*  “disadvantage is the application in practice” *(AUTHP1004 – aged 64 years)* |
| (8) Stakeholders’ views of the frequency of receiving summaries/predictions/advice from the LifeChamps digital platform | Frequency of predictions and advice | Other | "It would depend on the overall health of the person or needs of their support system at the outset, so for most people annually but maybe 6 monthly for others." *(UofGP21-aged 55 years)*  "It depends how accurate they are and how I would benefit from this information." *(UofGP37- aged 54 years)*  "At the end of treatment, with occasional perhaps every 3 months*" (UofGP36 – aged 63 years)*  “6-monthly” *(APCUKP3 – age 60 years)*  “When something changes” *(APCSWP1 – aged 50 years)*  “I do not know. Let's say 2 times per year.” *(APCSWP4 – aged 54 years)*  "Once a month or quarter, depending on the need" *(HULAFESP2 – aged 61 years)*  "As often as the patient requires." *(HULAFESP8 – aged 78 years)*  "I think that it is more important that the advice reaches people who are starting treatment, to avoid losing their strength and the desire to continue." *(HULAFESP9 – aged 66 years*) |
| (9a) Stakeholders’ expectations of health professionals’ actions when using the LifeChamps digital platform | Cancer survivor expectations of healthcare professionals’ actions | Communication | "be available for an in-depth discussion rather than be fobbed off with a 10 min appointment" *(UofGP39 - aged 54 years)*  "Better communication" (*HULAFESP4 – aged 63 years)*  "To collaborate by evaluating them and encouraging their use if they consider them appropriate." *(HULAFESP5 - aged 67 years)* |
|  |  | Adjust follow-up care | “To discuss with me and adapt the data to my own health problem.” *(AUTHP1010- aged 52 years)*  “To use for assessment and treatment” *(APCSWP4 – aged 54 years)* |
| (10) Comfort with the technology suggested for the LifeChamps digital platform | Cancer survivor comfort with technology | Barriers reported | “I have not learned to send an SMS for so many years that I have a mobile phone… Maybe it adds some stress to me, I do not know, and I do not want to know.” *(AUTHP0003 – aged 75 years)*  "I don't usually use them, better personalized attention" *(HULAFEP8 – aged 78 years)* |
