# Supplementary material for: A multinational investigation of healthcare needs, preferences, and expectations in supportive cancer care: co-creating the LifeChamps digital platform
Source: J Cancer Surviv. 2022 Nov 11;17(4):1094–110. doi: 10.1007/s11764-022-01289-7 (PMC9650169; doi:10.1007/s11764-022-01289-7)
Supplement: Supplementary file 4 — Supplementary file4 (DOCX 21 KB) [file 11764_2022_1289_MOESM4_ESM.docx]

Online Resource 4: Illustrative quotes of family caregivers’ perspectives of survivorship support, and the developing LifeChamps digital platform

| Shared Theme | Theme | Coded category | Illustrative quote |
| --- | --- | --- | --- |
| (1) Stakeholders’ priorities for cancer survivorship | Family caregivers’ own priorities and experiences since supporting the survivor | Family life  Psychological/emotional | “My priority is my mother who I take care of because she has not yet  finished with the side effects of her surgery” *(AUTHGR1018- FC aged 27 years, patient age unknown)*  "It’s a worry for us. I mean, obviously, for any...my husband, it’s his brother, he’s got cancer and he seems well. Every time he goes for treatment, you worry" *(UofG34)* |
| (2) Stakeholders’ health concerns /needs relating to age | Family caregiver health needs/concerns relating to the age of survivor | Psychological/emotional | "He needs ongoing emotional support, both for his own recovery and even more particularly given my diagnosis" *(UofG41 – FC aged 62 years, patient aged 61 years)*  "Feeling comfortable with her body after a mastectomy" *(UofG65 – FC aged 62 years, patient aged 55 years)* |
| (3) Stakeholders’ experiences of support or information provision | Survivor needs for support | Psychological/emotional | “Definitely psychological to accept the change he is experiencing and the limitations he now has in his life.” *(AUTHGR1016 – FC aged 25 years, patient specific age unknown)*  "Because of the depression caused by having cancer, her emotional life is very sad (she never wants to do anything) and she is always (almost) depressed" *(HULAFESP11 – FC aged 58 years, patient aged 82 years)* |
| (4) Stakeholders’ views on family support during survivorship | Family caregivers’ need for support  Cancer survivor support | Psychological/emotional | "emotional support need is ongoing" *(UofG41- FC aged 61 years, patient aged 62 years)*  "courage and desire to live" *(HULAFESP14 – FC aged 48 years, patient aged 68 years)*  “had to deal with incontinence with pelvic floor exercises” *(AUTHGR0006 – FC aged 68 years, patient aged 72 years)* |
| (5) Stakeholders’ concerns due to Covid-19 | Family caregiver and survivor concerns due to Covid-19 | Psychological/emotional  Physical / symptom related | “the fear of coronavirus due to the vulnerable immune system” *(AUTHGR1019 – FC aged 35 years, patient age unknown)*  "We try not to get infected by going out on the street" *(HULFESP14 – FC aged 48 years, patient aged 68 years)* |
| (6) Stakeholders’ views on ideal health services and support in survivorship | Family caregivers’ views on ideal health services/support | Practical and day-to-day living | “Who is now there to help you on how to access services easily” *(UofG3- FC aged 61 years, patient age unknown)*  "Counselling" (UofG2 – FC aged 30 years, patient aged 64 years)  "Mental health services - how to adjust to a new normal?" (*UofG1-FC aged 45 years, patient age unknown)* |
| (7) Stakeholders’ perspectives and expectations of the LifeChamps digital platform | The developing LifeChamps platform | Positive / advantages  Critique / disadvantages | "Extra support is always good" *(HULAFESP12 – FC aged 55 years)*  "speed in diagnoses and possible treatments." *(HULAFESP10 – FC aged 56 years)*  “the disadvantage is that the system is not anthropocentric.” *(AUTHC1023 – FC aged 50 years)*  "The danger of treating ourselves as numbers, cattle, etc." *(HULAFESP10 - FC aged 56 years)*  "Being too aware of data about your health (hypochondria)" *(HULAFESP10 - FC aged 56 years)*  “Could predict issues that may not occur*” (UofG3 – FC aged 61 years)* |
| (8) Stakeholders’ views of the frequency of receiving predictions/advice from the LifeChamps digital platform | Frequency of predictions and advice | Other | “Even once a week I do not mind*” (AUTHC0005 – FC age unknown)*  “Regularly to be constantly updated on the situation*” (AUTHC1016 - FC age 25 years)*  "To the extent that it can affect the patient" *(HULAFESP11 – FC aged 58 years)*  "As often as each patient requires" *(HULAFESP14 – FC aged 48 years, patient aged 68 years)*  “Annually” *(UofG65 – FC aged 62 years, patient aged 55 years)*  “Annually, better 6-monthly” *(UofC41 – FC aged 62 years, patient aged 61 years)*  “Quarterly or maybe every couple of weeks*” (UofG3 – FC aged 61 years, patient age unknown)*  “No need if filtered through a health care professional” *(UofG1 – FC aged 45 years)* |
| (9a) Stakeholders’ expectations of health professionals’ actions when using the LifeChamps digital platform | Expectations of health professionals’ actions | Adjust follow-up care | "immediate action in the event of a problem in this regard." *(HULAFESP10 – FC aged 56 years, patient aged 61 years)*  "Use them and not remain as a mere report" *(HULAFESP11 -FC aged 58 years, patient aged 82 years)* |
| (10) Comfort with the technology suggested for the LifeChamps digital platform | Comfort with technology | Positive / advantages | “Absolutely familiar” *(AUTHC1021 -FC aged 51, patient age unknown)*  "Overall comfortable" *(HULAFESP10 – FC aged 56 years, patient aged 61 years)*  "Very good, I use them every day" *(HULAFESP11- FC aged 58 years, patient aged 82 years)*  "I don't usually have problems with these devices*" (HULAFESP14 -FC aged 48 years, patient aged 68 years)*  “I use my phone for everything, but it used to be computers for everything, and my iPad, I hardly ever open now, it’s the phone because it’s there all the time." *(UofGC34 – FC aged 62 years, patient aged 64 years)* |
