# Supplementary material for: A multinational investigation of healthcare needs, preferences, and expectations in supportive cancer care: co-creating the LifeChamps digital platform
Source: J Cancer Surviv. 2022 Nov 11;17(4):1094–110. doi: 10.1007/s11764-022-01289-7 (PMC9650169; doi:10.1007/s11764-022-01289-7)
Supplement: Supplementary file 5 — Supplementary file5 (DOCX 38 KB) [file 11764_2022_1289_MOESM5_ESM.docx]

Online Resource 5: Illustrative quotes on healthcare professionals’ perspectives on supporting cancer survivors, and on the developing LifeChamps digital platform.

| Shared Theme | Healthcare professional Theme | Coded category | Illustrative quote |
| --- | --- | --- | --- |
| (1) Stakeholders’ priorities for cancer survivorship | Healthcare professionals’ priorities for support | Best supportive care | "Function, symptom management, fitness / stamina affects meaningful activity, identifying meaningful goals" *(UofGHP12)*  "advice about diagnosis, management and follow up. Ensure that planned follow up takes place in a timely fashion. Often patients do not take in or remember all the information they are given at hospital." *(UofGHP14)*  “psychological support” *(APCSW1)*  “easy to access, everyday help” *(APCSW8)*  “Treatment of treatment complications, close monitoring of the disease for possible relapses, interdisciplinary discussions in difficult-complicated cases.” *(AUTHGR1032)*  “Facilitation with appropriate arrangements of visits, effort to negotiate with colleagues to avoid unnecessary travel” *(AUTHGR1040)*  “My team includes a psychologist and a nutritionist, so we try to give them at least 2 of the 3 factors related to lifestyle nutrition and psychology, the exercise part is missing. Clearly trying to keep reassuring them that this is a chronic illness that no one has to deal with like other chronic illnesses. The effort to adapt the observation to the personality of everyone. (...) put them in a holistic monitoring program” *(AUTHGR0010)* |
|  |  | Frequent monitoring, follow up and communication | “to be careful and to pay attention even to slight symptoms that may have relation to cancer-recurrence" *(APCSW10)*  “to identify sign for new tumor or recurrence” *(APCSW13)*  “availability important” *(APCSW2)*  “Continuous monitoring of the patient's clinical picture and examinations.” *(AUTHGR1026)* |
|  |  | Survival | "The priority is to know the level of quality of life and perception of their health and to verify that there is no alteration." *(HULAFESP1)*  "The priority in the middle-aged patient is to cure him and secondly to avoid the comorbidity of the treatment." *(HULAFESP2)*  "In the case of the elderly (...) our objective is to offer the treatment that best adapts to their state of health and therefore offers the best life expectancy with the highest possible quality of life. " *(HULAFESP3)* |
| (2) Stakeholders’ health concerns/needs relating to age | Perspectives on cancer survivors post-treatment health needs | Psychological support | "Middle-aged patients (….) frequently, alterations in mood." *(HULAFESP3)*  “sleep disturbance. Worry and stress” *(APCSW11)*  “the patients are worried to relapse” *(APCSW6)*  “lack of sociability, fear of commentary, psychological, sociability” *(AUTHGR1029*)  “[They] experience anxiety and distress for possible relapse, need for support and understanding that the situation is under control” *(AUTHGR1042)* |
|  |  | Management of physical symptoms | "Fatigue, muscle atrophy, deconditioning, diminished balance control" *(UofGHP1)*  “...joint project (Improving Cancer Journey Team) by Macmillan and Glasgow City Council, kind of tied in with the Macmillan long term conditions team and some of the allied support networks, they basically offer a holistic health needs assessment whenever it’s requested. ...it can be done at the end of treatment or at any point in the future." *(UofGHP4)*  "Many of them (usually over 65 years old) had fragility and loss of autonomy due to insecurity as well as physical and mental limitations," *(HULAFESP1)*  "Middle-aged patients (…) Sometimes they also report chronic osteoarticular pain" *(HULAFESP3)*  “loss of sensation - disturbance in feet” *(APCSW7)*  “rehabilitation at home after treatment” *(APCSW8)*  “side effects” *(APCSW5)*  “Need to relieve post-cancer pain, other complications and desire to return to daily activities.” *(AUTHGR1027*)  “fatigue, treatment of side effects” *(AUTHGR1041)* |
| (3) Stakeholders’ experiences of support or information provision | Views on age-related support for patient/family post-treatment | Practical and day-to-day living | "I would like to see a reduction in assuming the patients are aware of the role and benefits of increasing PA and being part of their rehab." *(UofGHP1)*  "Need better access to exercise programs and to look at other lifestyle changes that could promote physical and emotional wellbeing." *(UofGHP21)*  “Carer support, lifestyle education including fatigue management/managing breathlessness/cognitive rehab, advice re keeping fit and active” *(UofGHM10)*  “nutritional” *(AUTHGR1042)* |
|  |  | Psychological support | “emotional support, attention to minimizing changes in body image produced by the treatment, aids for progressive return to work” *(HULAFESP3)*  “Those patients who came to post-treatment control and came to coincide with them (which were very few), the support was psycho-social knowing that you can count on the healthcare team whenever you need” *(HULAFESP1)*  “follow up...psychologically important” *(APCSW8)*  “need can be both physical and psychological” *(APCSW1)* |
|  |  | Management of physical symptoms | “to come earlier for rehab in order to avoid patient's loss of movement, physical condition and strength” *(APCSW11)*  “Contact nurse with possibility to contact in case of suspicion, concern or reassurance” *(APCSW13)*  “need can be both physical and psychological” *(APCSW1)* |
|  |  | Information | “The support that was offered to those included in post-treatment follow-up, was the total connection in case of needing information. For example: in the covid period, many patients in the EoS period called to find out if they were at risk or not and if they could go to work quietly.” *(HULAFESP1)*  “In both middle-aged and elderly patients, we need time to be able to clearly explain the effects of the treatment and its expectations, to be able to investigate those adverse effects that the patient may not be aware of because they are not related to the treatment.” *(HULAFESP2)*  “they have a lot of questions, they have information gaps for the continuation of their monitoring too, especially the older people” *(AUTHGR0008)*  “Proper information about possible complications after surgery and subsequent radiotherapy or chemotherapy.” *(AUTHGR1027)* |
| (5) Stakeholders’ concerns due to Covid-19 | Views on cancer survivors’ concerns due to Covid-19 | Experiences with health access | "dramatic reductions in elderly patients attending clinics and having treatment" *(UofGHP3*)  "Levels of support are less, especially face to face support. Older people are less able to use video consultations and may not hear well on the telephone. Communication can be more challenging." *(UofGHP14)*  "anxiety about attending hospital and their increased risk of contracting COVID-19" *(UofGHP30)*  "In addition, the collapse in the clinical hospital in the area of diagnostic imaging did not help, because many of our requests for CT, mammography, nuclear medicine and ultrasound were postponed or canceled by covid (many times without notifying the patient or the nurse of the patient." *(HULAFESP1)*  “important decisions such as chemotherapy have been made through the telephone” *(APCSW10)*  “delayed diagnosis.” *(APCSW13)*  “fear of visiting hospitals resulting in inadequate monitoring and non-compliance with appropriate protocols” *(AUTHGR1042)* |
|  |  | Experiences with daily living and community care | “More lonely” *(APCSW9)*  “Worried to meet their relatives. More remote contacts” *(APCSW10)*  “patients with undergoing cancer rehabilitation are maybe extra careful.” *(APCSW11) “*Isolated, feeling helpless, away from doctors and scared as a high-risk group” *(AUTHGR1036)* |
|  |  | Experiences with follow up services | Yes, in general the elderly patient has stopped coming for consultations, has lost follow-up, has delayed treatment, has not manifested his discomfort and his health condition has worsened." *(HULAFESP2)*  “Fear and non-compliance with follow-up visits and follow-up examinations.” *(AUTHGR1033)*  “There are patients who delay attending the doctor, or skip visits or postpone surgeries for fear of COVID-19 in hospitals” *(AUTHGR1040)* |
| (6) Stakeholders’ views on ideal health services and support in survivorship | Views on health care services post-treatment | Primary care services | "Community teams to provide practical support and assist in maintaining independence." *(UofGHP3)*  "Specialist cancer care nurses are undoubtedly the best contact for both patients, relatives and primary care.” *(UofGHP22)*  “A holistic provision of health care addressing not only physical issues like medication side effects and disease symptoms but assessing emotional and social needs. This should be close to the patient in their community and linked with their primary care provider" *(UofGHP34)*  “regular monitoring by a support team, which will detect the possible progression of the disease and will refer accordingly that there is a certain health professional who monitors the observance of the monitoring protocol” *(AUTHGR1042)* |
|  |  | Hospital follow up services | "psycho-oncology, a liaison nurse with a social worker and home care unit," *(HULAFESP1)*  "psychological care, rehabilitation or physical therapy, endocrinology" *(HULAFESP3)*  “specialized holistic care centre” *(AUTHGR1041)* |
|  |  | Remote monitoring services | “telephone number that they can ring to their oncologist-cancer nurse” *(APCSW8)*  “Contact nurse first priority” (*APCSW13)* |
|  |  | Home care services | “There must be flexibility. Some do not need anything. Some people need someone to help them with even the simplest things (bathroom, toilet)” *(AUTHGR1037)*  “home help, health guests, home monitoring by post-care teams” *(AUTHGR1040)* |
| (7) Stakeholders’ perspectives and expectations of the LifeChamps digital platform | Perspectives on the LifeChamps system | Praise / advantages | "I would be very interested in using this kind of technology it is the way forward. I think we could record patient’s activity very easily and this would encourage patients to improve their activity." *(UofGHP3)*  "Definitely need more work to look at needs of older patients. The use of multiple types of information and combining them could be good. Technology that does not rely on the user to gather data e.g., motion sensors could provide additional info." *(UofGHP21)*  "it could also enable patients themselves to be proactive in terms of accessing services or having a centralised or even personalised suggestions of how to address and how to improve their own quality of life. I think that would certainly be an advantage to try and get people more IT” *(UofGHP4)*  “Particularly useful - It will fill a huge gap in the most common cancer in men. To evaluate and discover the needs of patients!” *(AUTHHP1036)*  “will probably help to highlight the patients who need more attention. It is interesting to note the use of impersonal mass media to produce a personalized approach” *(AUTHHM1042)*  “to identify patients with greater need and type of need” *(APCHP13)*  “to bring to surface and make visible needs from a patient group that is not common (for rehabilitation)” *(APCHP11)*  “to improve healthcare through systematically observe and quantify the needs that exist” *(APCHP2)*  “auspicious for the purpose of developing such services” *(APCHP13)*  "to identify the unmet needs of the patient or family member that are as important as the pathology being treated, since in the end what we try to give the patient is a good state of health." (*HULAFEHP2)* |
|  |  | Critique / disadvantages | “There are many patients who still find IT technology difficult to management. At present the IT service within the health service is very outdated. To adapt to new technology improvements would need to be carried out" *(UofGHP23)*  "Need to reassure everyone that data was secure and obviously some folk may not have access to IT. Needs to be free for patients using mobiles (probs in past where calls are charged at premium rates for some NHS services)" (*UofGHP22)*  "its not gonna work. We need better follow up and care of patients, not a software to predict a failure that we will not have capacity to act upon. The NHS is already cluttered with multiple pieces of software that don't communicate." *(UofGHP29)*  “too many elderly patients do not have the ability, the electronic familiarity to be able to participate in such procedures […] The doctor does not have much time to be able to have this communication with the patient, it is impossible for the doctor to run it alone (…) maybe I will add users and trained secretaries [...] so it should be done in such a way so that non-doctors can handle it” *(AUTHHM0010)*  “I am considering if this can replace the complexity of the personal contact” *(APCHP10)*  “If the IT-system is not compatible it will be time consuming, ineffective, frustrating and can be unsafe for patient” *(APCHP12)*  “unnecessary” *(APCHP8)*  "the older patient may need more support to precisely express that information" *(HULAFEHP2)*  "As a disadvantage I think it will be to see if the accessibility is generalized. *" (HULAFEHP3)* |
| (7) Stakeholders’ perspectives and expectations of the LifeChamps digital platform | Expectations of LifeChamps system | Physical symptoms | “to be able to quickly get the measurement [e.g.] of bone mass from the radiology laboratory and the lipid measurements. If you add in this now the part of generic blood [exams] […] you have automatically reached a very good point for the rough stuff. An oximeter that measures oxygenation which should also give you the beats [...] and the oximeter data to be inserted [in the app] automatically” *(AUTHHM0010)*  “early indications” *(APCHP8)*  “alarm for suspected new tumor or recurrence” *(APCHP13)*  “Own suspicion for recurrence?” *(APCHP12)*  "Early indicators of: Lower urinary tract symptoms [and] Adverse effects of treatment" *(HULAFEHP2)* "accidental falls, weight, physical activity” *(HULAFEHP3)* |
|  |  | Emotional / psychological symptoms | “How they translate their own psychic world, their phobias, their anxieties, their sadness*” (AUTHHP0007*)  “to catch up signs for depression” *(APCHP2)*  “depressive factors” *(APCHP10*)  “Fear, depression? Need for support?” *(APCHP12)*  "and perception of health and quality of life." *(HULAFEHP1)* "Changes in health status and / or quality of life related to the pathology treated and or the treatment given" *(HULAFEHP2)* |
|  |  | Performance status and functioning | “clinical, laboratory and imaging monitoring depending on the type of tumour” *(AUTHHP1038)*  "It would analyse the lifestyle" *(HULAFEHP1)* "sincerity in compliance with the recommendations" *(HULAFEHP1)* "Degree of completion of the recommendations given" (H*ULAFEHP2)* "number of drugs taken by patients” *(HULAFEHP3)* |
|  |  | Practical and daily living | "exercise and collecting dietary information would be valuable. activity overnight would be helpful in providing care needs at home." *(UofGHP3)* "For patients the biggest things they will want to report/get answers to quickly is menopausal symptoms and strategies to manage these.” *(UofGHP11)*  "how they are managing the essential daily tasks of their lives depending on their ability - early indicators of these things becoming a struggle would allow intervention before they became impossible" *(UofGHP34)*  “recording patient expectations, realistic goal setting, monitoring progress, designing a new treatment plan, redefining goals” *(AUTHHM1042)* |
| (7) Stakeholders’ perspectives and expectations of the LifeChamps digital platform | Presentation of information | Summary report notification | “there could be a summary of what is considered important” *(AUTHHP0009)*  “a general overview” *(AUTHHP0007)* |
|  |  | Graph or chart notification | “It is best to have these recorded so that your doctor can see the entire chart so that he/she has a much safer picture of how you spent the [last] 6 months.” *(AUTHHM0010)* |
|  |  | Other | "Linked into Clinical Portal so it can be viewed by all involved in their care." *(UofGHP1)*  "Ideally electronically either via email or through one of the patient records systems that exist - would ideally not want to open another platform” *(UofGHP21)*  “The idea of an online dashboard looks great. Would need to integrate smoothly with existing clinical systems.” *(UofGHP40)*  “it must synchronize with the already existing journal system” *(APCHP12)*  “through the normal channel which is the journal” *(APCHP10)*  “the best would be in patient's electronic journal” *(APCHP2)*  "Have access to the developed platform." *(HULAFEHP1)*  "Mobile application." *(HULAFEHP2)*  "through the computerized medical record, or in an online platform with easy and fast access." *(HULAFEHP3)* |
| (7) Stakeholders’ perspectives and expectations of the LifeChamps digital platform | Requirements for implementation | Tackle workload / | “Someone to help with follow up to use the data appropriately and ensure follow-up care to help use the data productively” *(UofGHP9)* |
|  |  | human resource barriers | “training of staff who will use it” *(AUTHHP0008)*  “Time (always time) to address this new "portal" and deal with what coming through it. Would need to work out who addressed these issues as for early breast cancer patients/survivors this is not usually oncologists but is often breast care nurses. For metastatic patients (and I am not sure if you are suggesting this system for met patients) it would be oncology BCNs and oncologists.” *(UofGHP11)*  "Significant increase in the provision of AHP interventions within cancer services.” *(UofGHM10*)  “Automation so that it is not time consuming” *(AUTHHP1036)*  “workload” *(AUTHHP1032*)  “we will have to see how these people who will do this work will be rewarded, because you will have to hire someone if you do it, so enough people” *(AUTHHM0010)*  "Involvement by all components (patients, health workers, etc ...)" *(HULAFEHP1)* |
|  |  | Tackle infrastructure barriers | "Having time to be able to provide us with the information, have access to information platforms, have prepared hardware and enable us to handle it seem important requirements to me. " *(HULAFEHP2)*  “Existence of infrastructure, each patient to be registered in a specific care unit and to follow the individualized follow-up plan” *(AUTHHM1042)*  “If the IT-system is not compatible it will be time consuming, ineffective, frustrating and can be unsafe for patients” *(APCHP12)*  “a demanding journal system needs to be developed” *(APCHP11)*  “the data should be able to get transferred automatic” *(APCHP9)* |
| (8) Stakeholders’ views of the frequency of receiving predictions/advice from the LifeChamps digital platform | Frequency of information | Other | “When the patient is due their clinical review (varies according to their diagnosis etc). There is not time to be looking at these scores and assessments outside of the current review times” *(UofGHP21)* "This would depend if patient is still being followed up in hospital setting. Most patients are discharged at 5 years. Most of ongoing care should be shared with community." *(UofGHP23)*  "as soon as available - means info is available in "real time" for clinicians instead of a weekly or monthly "dump" *(UofGHP22)*  “It would depend on what we were doing with this information - it would be impossible to receive this information for every patient we see as we do not have the ability to keep patients on our caseloads indefinitely.” *(UofGHM10)*  “once every 3 months” *(AUTHHM1042)*  “3-4 times in the first year, 2-4 times the second year.” *(APCHP13)*  “in predefined time according to specific agreement” *(APCHP12)*  “when I see the patient?” *(APCHP10)*  "I would say that between control visits (if they are every 6 months, then after 3 months have a telephone control through a socio-psycho-physical-emotional assessment survey)." *(HULAFEHP1)*  "before each patient visit" *(HULAFEHP3)* |
|  |  | On demand | “Every time I evaluate a new patient” *(AUTHHP1027)*  “depends on the parameter… there are people who are very good in health and I do not need to zoom (in time), there are people who are fragile, they would be interested at some point to zoom” *(AUTHHM0010)* |
| (9b) Healthcare professionals’ views on the use of patient-reported outcomes via the LifeChamps digital platform | Views on PROMs | PROMs | “Most PROMS we have experience of are designed for clinical trials and not to benefit the patients. Their primary aim is to establish cost of a QALY for health economic analysis. FACT scores seem good in that they are shorter and focus on function." *(UofGHP21*)  "EQ-5D-5L and Godin Leisure Time Exercise questionnaire.” *(UofGHP1)*  "DASH” (self-report outcome measure of upper limb symptoms and function questionnaire) “Fatigue - Macmillan CaPASEF outcome measures" (Cancer Physical Activity Standard Evaluation Framework) *(UofGHP37)*  "MyCaw (My Concerns and Wellbeing Checklist), Godin Leisure-Time Exercise Questionnaire, EQ-5D-5L health questionnaire, FACIT Fatigue Scale. Locally developed Patient and Carer Experience (PACE) surveys are also regularly completed, and clinician feedback gathered." *(UofGHM10)*  “activity, lack of pain, quality of sleep, diet” *(AUTHGR1029*)  “The key is the evaluation of the quality of life, which is often what is required. Also effects that concern in addition to the disease the patient's daily life such as symptoms or anxiety” *(AUTHGR1032)*  “The scale related to discomfort, the scale that has to do with pain” *(AUTHGR0010)*  “patient's participation in social life compared before and after illness/psychological prosperity” *(APCSW11)*  “Patient experience varies depending on the patient. All the care the patient receives in the health care system is what concerns them many times...” *(AUTHGR1026)*  It is fundamental to listen to the patient, so both PROMS and PREMS should be systematized in our usual practice because I believe that it would result in a better treatment of the individual patient. Sometimes we forget that we treat a patient with a personal situation and an own experience, so we only consider that it is a patient with a cancer with a specific stage and that we must treat it with the appropriate treatment." *(HULAFESP2)*  "I see it important to computerize the health system, since there is a lot of information that is lost, as the patient does not write it down, so he/she does not comment on it to the health system and the overworked health worker cannot concentrate on writing down everything necessary to notify good PROMs and PREMs " *(HULAFESP1)* |
